# Changes in Endurance Performance in Young Athletes During Two Training Seasons

**DOI:** 10.1515/hukin-2015-0117

**Published:** 2015-12-30

**Authors:** Łukasz Tota, Marcin Maciejczyk, Ilona Pokora, Jerzy Cempla, Wanda Pilch, Tomasz Pałka

**Affiliations:** 1 Department of Physiology and Biochemistry, Faculty of Physical Education and Sports, University of Physical Education, Krakow, Poland; 2 Department of Physiology, the Jerzy Kukuczka Academy of Physical Education in Katowice, Poland

**Keywords:** aerobic performance, middle- and long-distance running, physical training, ventilatory threshold

## Abstract

The aim of the study was to assess changes in endurance performance in young runners (females and males) during two training seasons. It involved 19 male and 16 female athletes aged 15–17 specializing in track-and-field middle and long distances runs. The following parameters were measured three times during the training season: maximal oxygen uptake, running economy, and the level of the second ventilatory threshold. Training volume and intensity during each season were analyzed within an 8-week period prior to the exercise tests. The volume and intensity of training at various stages of preparation in both seasons were similar. During the first year of observation, significant improvements in relative volume of maximal oxygen uptake were reported both in female and male athletes. During the second training season, it was found that running economy improved both in women and men, with no changes in maximal oxygen uptake. The same (in terms of volume and intensity) endurance training carried out with young runners during two consecutive training seasons can result in different training effects.

## Introduction

Endurance can be defined as the capacity to sustain a given velocity or power output for the longest possible time (Jones and Cartner, 2000). The key parameters used to assess endurance performance include metabolic (ventilatory) thresholds, maximal oxygen uptake (VO_2max_), and running economy (RE) (Joyner and Coyle, 2008). The second ventilatory threshold (VT_2_) (respiratory compensation point) is an important indicator which strongly correlates with endurance performance (Iwaoka et al., 1988; Maciejczyk et al., 2014). Exceeding this threshold leads to the accumulation of lactate in blood, hyperventilation, and the development of decompensated metabolic acidosis. Maximal oxygen uptake reflects an individual’s maximal rate of aerobic energy expenditure, and most studies of endurance training have shown some increase with time of training; however, the optimal exercise volume and intensity for developing this parameter is not known (Jones and Carter, 2000). Running economy is typically defined as the oxygen demand for a given velocity of submaximal running, and is determined by measuring the steady-state consumption of oxygen and the respiratory exchange ratio (Saunders et al., 2004). Endurance training coupled with other training methods has been shown to improve RE in untrained and moderately trained subjects, with trained runners having better RE than their untrained counterparts (Saunders et al., 2004). To improve RE, endurance training is most often combined with strength training, training in the heat, or with altitude exposure (Saunders et al., 2004). However, the influence of endurance training alone on RE is ambiguous (Jones and Carter, 2000). For example, Lake and Cavanagh (1996) reported no significant improvement in running economy after 6 weeks of endurance training. Similarly, Brisswalter and Lagros (1994) showed that during one training season there were no substantial changes in RE. This may be because such training studies (typically of 6 to 12 weeks in duration) are too short to produce a measurable improvement in running economy, especially in individuals who are already trained. It may be speculated that good exercise economy is somehow related to the total volume of endurance training performed, since the best economy values are often found in older and more experienced athletes (Jones and Carter, 2000; Knechtle et al., 2012; Trappe, 2007).

Adolescence is marked by major changes in somatic characteristics and improvement in cardiorespiratory fitness. During this period, VO_2max_ values in boys remain relatively stable, but they decrease in untrained girls due to an increase in body fat (Bitar et al., 2000; Rowland, 2007), however, training may reduce body fat in both males and females (Ballor and Keesey, 1991). Although female athletes performing a number of sports tend to be heavier than reference populations, they also, in general, have lower body fat percentages (Baxter-Jones and Maffulli, 2002). The study conducted by Maliszewski and Freedson (1996) showed that when running at a constant speed, running economy of adolescents was worse than in adults. This is mainly due to the fact that in children the respiratory function is not fully developed (higher ratio of ventilatory volume to volume of oxygen consumed in children than in adults) (Maliszewski and Freedson, 1996). During both adolescence and adulthood, there are sex differences in running economy (Helgerud et al., 1990; Morgan et al., 1999). It seems that an improvement in VO_2max_, VT_2_, and/or RE may lead to better endurance exercise performance; however, at present, very little is known about the most effective training practices aimed at improving specifically the key parameters of aerobic fitness (Jones and Cartner, 2000). In particular, there are not enough long-term studies on the changes in endurance performance in adolescent athletes with relatively short training experience. The lack of longitudinal work limits our understanding of the volume of training needed in order to optimize biological changes and performance (Berg, 2003).

The purpose of this study was to evaluate the changes in variables which determine endurance performance (VO_2max_, VT_2_, RE) in young female and male athletes during two training seasons, as well as to identify the sex differences in the level of analyzed indicators in the considered period.

## Material and Methods

### Study design

The study project was approved by the Bioethics Committee at the Regional Medical Chamber in Krakow, Poland. The study was conducted according to the Declaration of Helsinki. The athletes and their parents/guardians were informed about the purpose and course of the study and provided their written consent for their children participating in the study. The subjects had valid medical certificates, which was a condition to take the cardiac stress test. The stress tests were carried out under the constant supervision of a sports medicine doctor.

The study was carried out six times during two training seasons, three times each season (test A1, B1, C1: season one; test A2, B2, C2: season two). The stress tests and somatic measurements were performed three times per training season: at the beginning of the preparation period (test A1 and A2), at the end of the preparation period (test B1 and B2), and at the end of the competitive period (test C1 and C2). The subjects performed two stress tests: i) an incremental test (to determine VO_2max_ and VT_2_) and ii) a submaximal test at constant intensity (6 min) with individually matched speeds corresponding to VT_2_ (assessment of running economy). Both stress tests were conducted with a one-week interval.

The study also analyzed the training loads applied for 8 weeks immediately before the stress tests (except for the first test). Both in the first and second training season, the preparation period took 22 weeks, while the competitive period lasted 10 weeks.

### Participants

The study involved a total of 35 subjects, including 19 male and 16 female athletes specializing in track-and-field middle and long distances runs. The average training experience was 5.2±1.5 years in male participants and 4.8 ±1.1 years in female participants. In the first test, the average age was 16.4±0.85 years for males and 16.4±0.82 years for females. The detailed description of the study subjects is presented in Table 1.

### Anthropometric measurements

Prior to each series of tests, body height (BH), body mass (BM), fat mass (FM), and lean body mass (LBM) were measured. Body composition was determined with the electrical bioimpedance technique, using the Jawon Medical body composition analyzer, model IOI 353 (Korea), while body height was measured using a Martin-type anthropometer (USA), with accuracy to 1 mm.

### Incremental test

During the incremental test, maximal oxygen uptake and the level of the second ventilatory threshold were determined. The test was performed on a treadmill (h/p Cosmos, Saturn, Germany) with 1° incline. The test began with a 4 min warm-up, during which a subject was running at a constant speed: men 9.0 km·h^−1^, women 8.0 km·h^−1^; next, the running speed was increased every 2 min by o 1.1 km·h^−1^ in men and 1.0 km·h^−1^ in women. When the heart rate started to approach the maximum (age predicted HR_max_=220-age), the running speed was kept at a constant level, while the load was increased with a treadmill incline by 1° every minute. The test lasted until the volitional exhaustion. The criteria for determining VO_2max_ were as follows: respiratory exchange ratio (RER) above 1.1 and a plateau in oxygen uptake despite the load increase. During the test, an ergospirometer (919 Medikro, Finland) was used to measure oxygen uptake (VO_2_), RER, pulmonary ventilation (VE), production of carbon dioxide per minute (VCO_2_), expiratory carbon dioxide concentration (%FECO_2_), and ventilatory equivalent ratio for carbon dioxide (VE/VCO_2_). The heart rate (HR) was measured using a heart rate monitor (Polar S610i, Finland).

The second ventilatory threshold was determined based on the changes in respiratory indicators during the incremental test. The criteria for determining VT_2_ were as follows: a) %FECO_2_ peaked and then was reduced; b) VE/VCO_2_ reached the minimum value and then increased; c) after VT_2_ was exceeded, a non-linear increase in minute ventilation was recorded (Bhambhani and Singh, 1985; Binder et al., 2008). Next, the researchers determined the running speed (running intensity) at which the subjects achieved VT_2_: at this speed, the athletes performed a submaximal exercise protocol for 6 min, during which running economy was measured.

### Running economy

Running economy was evaluated in the steady state of the submaximal run at the speed corresponding to VT_2_ achieved while running on a treadmill for 6 min. The running speed was tailored to each participant and matched the running speed (intensity of exercise) at the level of the second ventilatory threshold (Fletcher et al., 2009). The test was conducted at a 1° incline. During the test, VO_2_ and the HR were measured. In the present study, VO_2_ and the HR were presented as the average level measured in the steady state. Oxygen uptake was shown in absolute values (l·min^−1^), and relative to body mass (ml·kg^−1^·min^−1^). Running economy was calculated as the consumption of oxygen required to run 1 km and expressed in (ml·kg^−1^·km^−1^).

### Training

Analysis of endurance training (volume and intensity) was carried out for 8 weeks prior to the B1-C2 tests. Based on the incremental test results, in series A1 and A2, each athlete was assigned three metabolic zones: below VT_2_ (below HR at VT_2_), at VT_2_ (HR at VT_2_±3 b·min^−1^), and above VT_2_ (above HR at VT_2_).

Training was performed on a synthetic track (400 m) and off-road routes of known distances, which allowed to precisely record the total covered distance. The athletes kept training diaries where, after each training unit, they registered their distance and mean heart rate. The heart rate (intensity of each training (%HR_max_)) was controlled by the heart rate monitors (Polar RS400, Finland). Training volume was presented as the total covered distance (km) the subject managed to run during 8 weeks and the distance covered in the three metabolic zones (below, at, and above VT_2_). The distance ran in individual training zones is also shown as % of the total covered distance (Table 2).

### Statistical analysis

In order to assess the significance of differences between groups and of changes in the variable during the two-year training period, the multi-factor analysis of variance (ANOVA) with repeated measurements was used. The analysis entailed the influence of the following factors: group (sex) and variable changes due to training. In addition, the interaction between the analyzed factors (gender and training) was evaluated. The homogeneity of variance within the groups was tested with the Levene’s test (the variance of analyzed parameters was similar in both groups). If the analysis of variance showed a significant change (*p*<0.05), the post-hoc analyses were carried out in order to evaluate the statistical significance of changes between different test series (Tukey HSD test). Detailed results of the post-hoc comparisons are presented in the tables. *performance in young athletes during two training seasons*

## Results

During the two-year training period, the male and female athletes’ body mass, lean body mass, and body height increased significantly. No significant changes were observed in body fat mass in women or men. However, there were significant sex differences in somatic characteristics (Table 1).

### Training analysis

At every stage of preparation, a completed training session within 8 weeks before exercise tests in the first and second year had a similar intensity both for males and females: about 70% of the training volume was conducted with an intensity similar to the corresponding VT_2_, 15–20% with an intensity above VT_2_, and 10–15% with an intensity below VT_2_ (Table 2).

During a period of 8 weeks before each stress tests, the volume of training in the first and second year was similar (i.e. B1 vs. B2 and C1 vs. C2). Important changes were found in total training volume during the considered period (F=9.74; *p*=0.00) as well as significant differences between groups (F=58.6; *p*=0.00) in the total volume of training. There were significant differences between the sexes in the volume of training sessions carried out at intensities: at, below and above VT_2_.

The results of post-hoc analyses showed that total volume of the distance ran by an athlete varied significantly between the various stages of the season, and also that at every stage of preparation, there were significant intergroup differences in the volume of training (Table 2).

### Changes in endurance performance during the two years of training

During the two training seasons, no improvement in running speed was observed at VT_2_ (F=0.79; *p*=0.55) for neither group, and in the next series of tests, VT_2_ was achieved at similar (F=0.69; *p*=0.63) intensity of about 78% VO_2max_. During the two-year training period there was a substantial rise in relative values of VO_2max_ (F=3.57; *p*=0.04). The post hoc analysis showed that significant changes in VO_2max_ were observed in both groups only during the first training season; in the second season, VO_2max_ stabilized (Table 3). During the studied period, the researchers recorded a statistically significant (F=2.93; *p*=0.02) improvement in running economy in both groups. The post hoc analysis also proved that a significant improvement in RE occurred only during the second season, while training conducted in the first year did not significantly influence RE (Table 4).

### Sex differences

Running speed at VT_2_ was significantly lower in women than in men (F=17.4; *p*=0.00). Statistically significant differences between female and male athletes were recorded in each test series in relative values of VO_2max_ (F=43.2; *p*=0.00) and in absolute (F=36.4; *p*=0.00) and relative (F=59.3; *p*=0.00) values of submaximal VO_2_ (Tables 3 and 4). There were no statistically significant (F=0.08; *p*=0.94) sex differences in RE in any of the test series (Table 4). The analysis of distinctive endurance performance did not indicate any relevant interactions between the analyzed main effects (sex and training).

## Discussion

The main aim of this study was to determine the impact of two-year endurance training on the following variables: maximal oxygen uptake, the second ventilatory threshold, and running economy. Our study showed that, in adolescent athletes, the same volume and intensity of training, carried out during an 8-week period before exercise tests, may lead to different training effects, depending on age of the subjects. In the first year of the study (age 16.5–17), training primarily improved the level of maximal oxygen uptake (about 6% in males and 8% in females). Similar training carried out in the second season (age 17.5–18) contributed significantly to the improvement of running economy in both sexes. Furthermore, in the second training season, there was a consistently high level of VO_2max_ (similar to the one recorded at the end of the first season). It can therefore be inferred that the effects of similar training (the same volume and intensity) performed over 8 weeks prior to the exercise tests, depend on athletes’ age: first, there was an increase in VO_2max_, followed by an improvement in running economy with the same high level of VO_2max_. It seems that such a course of training effects is beneficial. There are numerous factors limiting VO_2max_ and affecting the ability to increase this variable (Basset and Howley, 2000). Thus, improving endurance performance (in running) can be achieved by improving running economy and increasing the running speed at VT_2_. According to our knowledge, these results are extremely valuable, as this type of long-term observations are very rare. Previous studies typically had analyzed the impact of several weeks of physical training, and their results indicated that several weeks of training influenced endurance performance in different ways (Jones and Carter, 2000).

The results obtained in this study show that, in both seasons and at each stage, running economy was similar in men and women. Our results are contradictory to the results presented to date which prove significant differences in running economy between prepubertal boys and girls (Morgan et al., 1999), as well as adult men and women (Helgerud et al., 1990). Moreover, the female runners studied by Helgerud et al. (1990) achieved greater volume than the men, and yet their running economy was worse.

In our study, running economy was not evaluated at the same submaximal running speed for all the subjects, but with individually tailored running speed with intensity corresponding to the second ventilatory threshold. RE is usually assessed at the same speed for all study participants (Foster and Lucia, 2007), but the evaluation of RE at the second ventilatory threshold in athletes is far less performed (Mooses et al., 2013). Fletcher et al. (2009) argue that this is the right way to measure RE. Comparing different athletes at the same absolute running speed does not take into account the differences in speed associated with the ventilatory threshold or the differences in substrate utilization associated with differences in intensity relative to VO_2max_ (Fletcher et al., 2009).

The tested athletes (both women and men) were not economical. RE in the athletes aged about 20, specializing in track-and-field middle and long distance runs, measured with oxygen uptake required to run 1 km ranged from 197 ml·kg^−1^·km^−1^ to 207 ml·kg^−1^·km^−1^ (Bragada et al., 2010). During all test series, the values of the energy cost of running obtained in our study significantly exceeded the previously specified level of 200 ml·kg^−1^·km^−1^. This proves that there is a significant possibility for improvement in RE in the studied athletes.

Running economy is closely linked to body dimensions and composition (Saunders et al., 2004). The study participants were still adolescents. During the two-year period of training, their body height, body mass and lean body mass changed substantially. Previous studies had shown that individuals with smaller body size had better running economy than subjects who were taller and heavier (Foster and Lucia, 2007). Of particular importance is body fat mass: an increase in body fat requires muscles to make extra effort to move (accelerate) the lower limbs during the run, which can lead to an increase in energy expenditure (Arrese and Ostáriz, 2006). Body fat of the studied female and male athletes remained at a similar level, which probably did not affect RE in the next series of tests. Significant changes in body size during puberty may affect biomechanics of movement (co-ordination), which in turn may also significantly influence running economy (gait patterns, stride length, etc.) (Anderson, 1996). Mooses et al. (2013) indicated that in level distance runners RE was influenced by specific training rather than by body composition variables.

The results of this study are important for track and field coaches working with youth athletes. Our long-term observations indicate that the same (in terms of volume and intensity) endurance training in runners may result in different training outcomes in two consecutive training seasons (when athletes grow older). No significant differences were found in running economy between male and female subjects; and the differences in the level of VO_2max_ were similar to the previously presented data (Saltin and Astrand, 1967). The results of our study should be taken into account by coaches when planning training loads for youth athletes specializing in track-and-field middle and long distance runs.

## Figures and Tables

**Table 1:** Age and the level of somatic variables in male and female subjects before each test series

Season		1			2			Post hoc (*p*)

Measurement		A1	B1	C1	A2	B2	C2	
Age (y)								
♂	χ̄ SD	16.5±0.9	16.9±0.9	17.1±0.9	17.5±0.9	17.9±0.9	18.1±0.9	
♀	χ̄ SD	16.5±0.8	16.9±0.8	17.1±0.8	17.5±0.8	17.9±0.8	18.1±0.8	
BM (kg)								
♂	χ̄ SD	62.0±7.9	62.8±7.7	62.4±7.1	64.7±6.3	64.7±6.0	63.8±6.4	A1-C2 (0.00)
♀	χ̄ SD	51.9±5.4	52.3±5.1	50.7±5.0	52.5±5.4	53.6±5.5	52.9±4.9	A1-C2 (0.00)
	*p*	0.00	0.00	0.00	0.00	0.00	0.00	
LBM (kg)								
♂	χ̄ SD	56.9±7.0	57.2±6.9	57.3±6.2	59.2±5.6	59.2±5.6	58.2±5.9	A1-C2 (0.00)
♀	χ̄ SD	45.0±7.2	45.0±6.9	43.4±5.0	44.0±5.4	45.7±6.1	45.0±5.3	A1-C2 (0.00)
	*p*	0.00	0.00	0.00	0.00	0.00	0.00	
FM (kg)								
♂	χ̄ SD	5.0±1.3	5.9±1.8	5.1±1.5	5.4±1.3	5.5±1.2	5.5±1.8	NS
♀	χ̄ SD	8.1±1.2	8.5±1.3	8.5±1.6	9.0±1.6	8.5±1.2	8.5±1.4	NS
	*p*	0.00	0.00	0.00	0.00	0.00	0.00	
BH (cm)								
♂	χ̄ SD	173.8±5.8	174.5±5.2	174.9±4.8	175.4±4.6	175.6±4.2	175.6±4.4	A1-C2 (0.00)
♀	χ̄ SD	163.9±4.2	164.6±3.9	164.8±3.9	165.0±3.8	165.8±3.9	166.0±4.0	A1-C2 (0.00)
	*p*	0.00	0.00	0.00	0.00	0.00	0.00	

1,2 - number of the season; A1 and A2 - measurement at the beginning of the preparation period in season 1 and 2; B1 and B2 - measurement at the end of the preparation period in season 1 and 2; C1 and C2 - measurement at the end of the competitive period 1 and 2; BH – body height; BM – body mass; LBM – lean body mass; FM – body fat mass; NS – differences not statistically significant (p>0.05)

**Table 2:** Total distance and the distances covered in three metabolic zones (below, above, and at VT_2_) during 8 weeks of training before particular exercise series

Season		1		2			Post hoc (*p*)

Test		B1	C1	A2	B2	C2	
Total distance covered (km)							
♂	χ̄ SD	489.2±54.1	456.3±60.1	384.2±77.2	490.4±65.1	420.3±51.5	B1-C1 (0.04) A2-B2 (0.00) B2-C2 (0.04)
♀	χ̄ SD	371.1±56.0	324.3±49.3	317.1±62.1	408.6±55.5	350.6±56.7	B1-C1 (0.04) A2-B2 (0.04) B2-C2 (0.04)
	*p*	0.00	0.00	0.00	0.00	0.00	
Distance covered at VT_2_ intensity (km)							
♂	χ̄ SD	359.0±59.2	323.7±90.0	300.8±83.0	339.2±64.2	309.7±43.0	
	[%]	73.1	70.1	77.4	68.8	73.9	
♀	χ̄ SD	260.8±45.9	230.1±42.7	251.1±74.3	300.1±50.9	265.1±54.3	
	[%]	70.3	70.7	77.8	73.2	75.4	
	*p*	0.00	0.00	0.00	0.00	0.00	
Distance covered below VT_2_ intensity (km)							
♂	χ̄ SD	60.1±25.0	35.6±14.0	33.3±13.3	72.8±18.2	23.2±14.0	
	[%]	12.5	7.9	9.1	15.1	5.5	
♀	χ̄ SD	50.3±26.2	25.9±10.3	20.9±8.6	49.7±16.2	14.5±10.4	
	[%]	13.1	8.2	7.1	12.5	4.3	
	*p*	0.00	0.00	0.00	0.00	0.00	
Distance covered above VT_2_ intensity (km)							
♂	χ̄ SD	70.1±10.8	97.0±18.7	50.1±15.7	78.4±13.0	87.4±25.6	
	[%]	14.4	22.0	13.5	16.1	20.7	
♀	χ̄ SD	60.0±14.3	68.3±16.7	45.1±24.8	58.8±17.9	71.1±24.5	
	[%]	16.6	21.1	15.1	14.3	20.3	
	*p*	0.00	0.00	0.00	0.00	0.00	

1,2 - number of the season; A2 - measurement at the beginning of the preparation period in season 2; B1 and B2 - measurement at the end of the preparation period in season 1 and 2; C1 and C2 - measurement at the end of the competitive period 1 and 2; VT_2_ - second ventilatory threshold; [%]: percentage of total distance covered

**Table 3:** Maximal oxygen uptake (VO_2max_), maximal heat rate (HR_max_) and intensity at the second ventilatory threshold (%HR_max_, %VO_2max_) registered in the incremental test

Season		1			2			Post hoc (*p*)
Test		A1	B1	C1	A2	B2	C2	
VO_2max_ (ml·kg^−1^ ·min^−1^)								
♂	χ̄ SD	63.8±5.2	65.2±5.2	67.7±3.6	66.7±7.0	65.8±5.4	66.3±6.2	A1-C1 (0.04)
♀	χ̄ SD	54.6±5.7	58.6±7.8	58.9±6.0	59.4±5.9	59.4±5.4	58.4±4.4	A1-C1 (0.04)
	*p*	0.00	0.00	0.00	0.00	0.00	0.00	
HR_max_ (b·min^−1^)								
♂	χ̄ SD	201.4±5.9	199.5±6.9	199.3±6.0	199.0±7.2	199.0±7.1	199.4±5.5	NS
♀	χ̄ SD	194.1±6.4	193.8±6.7	195.4±7.4	193.9±7.3	194.2±7.3	195.5±8.1	NS
	*p*	NS	NS	NS	NS	NS	NS	
%HR_max_								
♂	χ̄ SD	84.7±2.3	84.8±5.5	85.6±3.6	85.4±4.6	82.8±4.7	85.8±4.9	NS
♀	χ̄ SD	88.7±4.5	88.2±3.2	85.0±5.9	89.1±4.1	87.0±4.2	85.4±2.5	NS
	*p*	NS	NS	NS	NS	NS	NS	
%VO_2max_								
♂	χ̄ SD	77.3±8.6	80.0±10.3	79.1±6.0	77.4±9.3	77.9±7.5	78.0±10.1	NS
♀	χ̄ SD	78.8±7.8	76.4±13.0	76.1±9.2	79.3±14.1	78.3±10.9	78.4±9.6	NS
	*p*	NS	NS	NS	NS	NS	NS	

1,2 - number of the season; A1 and A2 - measurement at the beginning of the preparation period in season 1 and 2; B1 and B2 - measurement at the end of the preparation period in season 1 and 2; C1 and C2 - measurement at the end of the competitive period 1 and 2; NS – differences not statistically significant (p>0.05)

**Table 4:** Running speed (v), heart rate (HR), oxygen uptake (VO_2_), running economy (RE) and exercise intensity (%VO_2max_; %HR_max_) registered in the steady state of the submaximal run

Season		1			2			Post-hoc (*p*)
Test		A1	B1	C1	A2	B2	C2	
v (km·h^−1^)								
♂	χ̄ SD	12.4±1.3	12.8±1.1	12.7±0.8	12.2±1.0	12.5±1.3	13.2±1.1	NS
♀	χ̄ SD	10.4±1.2	10.8±0.8	10.8±0.8	10.9±1.3	11.0±0.8	11.0±0.8	NS
	*p*	0.00	0.00	0.00	0.00	0.00	0.00	
VO_2_ (l·min^−1^)								
♂	χ̄ SD	3.00±0.5	3.26±0.5	3.37±0.4	3.30±0.5	3.29±0.4	3.41±0.4	A1-C1 (0.01) A1-A2 (0.00) A1-B2 (0.01) A1-C2 (0.00)
♀	χ̄ SD	2.23±0.4	2.39±0.3	2.24±0.4	2.47±0.5	2.43±0.2	2.34±0.2	NS
	*p*	0.00	0.00	0.00	0.00	0.00	0.00	
RE (ml·kg^−1^·km^−1^)								
♂	χ̄ SD	243.4±19.4	249.8±14.2	256.9±18.7	256.1±21.2	248.6±19.8	246.7±20.1	A2-C2 (0.01)
♀	χ̄ SD	250.2±19.3	249.8±20.2	249.4±20.7	266.2±21.8	254.1±10.0	246.9±15.9	A2-C2 (0.01)
	*p*	NS	NS	NS	NS	NS	NS	
VO_2_ (ml·kg^−1^·min^−1^)								
♂	χ̄ SD	49.1±4.8	51.9±4.7	53.6±3.3	51.1±5.9	51.4±4.4	53.5±5.1	NS
♀	χ̄ SD	42.6±4.1	44.6±3.8	44.6±3.7	47.5±7.5	46.5±4.3	47.2±5.7	NS
	*p*	0.00	0.00	0.00	0.00	0.00	0.00	
%VO_2max_								
♂	χ̄ SD	77.0±9.1	79.6±8.8	79.2±7.1	76.6±8.5	78.1±7.9	80.7±8.2	NS
♀	χ̄ SD	78.0±8.3	76.1±11.8	75.7±7.3	80.0±10.8	78.3±9.7	80.8±8.1	NS
	*p*	NS	NS	NS	NS	NS	NS	
HR [b·min^−1^]								
♂	χ̄ SD	171.7±6.8	170.9±6.8	172.1±6.2	170.7±5.5	171.3±3.3	172.2±7.4	NS
♀	χ̄ SD	172.8±9.8	171.6±6.2	170.2±8.5	173.4±6.5	172.2±6.3	170.9±4.7	NS
	*p*	NS	NS	NS	NS	NS	NS	
%HR_max_								
♂	χ̄ SD	85.3±3.6	85.7±4.1	86.4±4.5	85.8±3.7	86.1±4.3	86.4±3.9	NS
♀	χ̄ SD	89.0±4.1	88.5±4.4	87.1±3.9	89.4±4.6	88.7±4.0	88.0±4.1	NS
	*p*	NS	NS	NS	NS	NS	NS	

1,2 - number of the season; A1 and A2 - measurement at the beginning of the preparation period in season 1 and 2; B1 and B2 - measurement at the end of the preparation period in season 1 and 2; C1 and C2 - measurement at the end of the competitive period 1 and 2; NS – differences not statistically significant (p>0.05)
